# Nucleolar small molecule RNA SNORA75 promotes endometrial receptivity by regulating the function of miR-146a-3p and ZNF23

**DOI:** 10.18632/aging.203007

**Published:** 2021-05-24

**Authors:** Peng Wei, Haitao Wang, Yuebai Li, Ruixia Guo

**Affiliations:** 1 Department of Gynaecology and Obstetrics, The First Affiliated Hospital of Zhengzhou University, Zhengzhou 450052, People's Republic of China; 2 Department of Orthopedic Surgery, The First Affiliated Hospital of Zhengzhou University, Zhengzhou 450052, People's Republic of China; 3 Department of Biochemistry and Molecular Biology, School of Basic Medical Sciences, Zhengzhou 450052, People's Republic of China

**Keywords:** SNORA75, miR-146a-3p, ZNF23, receptivity, endometrial

## Abstract

Endometrial receptivity enables the embryo to attach, invade and develop, forming a new individual and species continuity. Small nucleolar RNAs (SnoRNAs) are a class of non-coding RNAs comprising two classes: C/D box snoRNAs and H/ACA box snoRNAs. Aberrant expression of SNORNAs has been reported in tumorigenesis. However, the role of SNORNAs in maintaining endometrial receptivity has not been reported. First, we detected SNORNA expression in endometrial tissues during proliferative and secretory endometrial periods using RNA sequencing. SNORA75 expression was higher in the secretory endometrium, and its overexpression significantly promoted the proliferation, migration and invasion of endometrial cells. The results of analysis with bioinformatics software and RNA pulldown experiments showed that miR-146a-3p interacted with SNORA75. Western blotting showed that miR-146a-3p regulated the expression of ZNF23, whose overexpression significantly promoted the proliferation, migration and invasion of endometrial cells. SNORA75 modulates endometrial receptivity through the miR-146a/ZNF23 signaling pathway.

## INTRODUCTION

The ability to accept normal endometrial implantation as the basis for normal pregnancy is called receptivity. Endometrial receptivity is a complex process that allows embryos to attach, invade and develop, eventually forming a new individual and continuing the species. Endometrial receptivity is a time limit for endometrial epithelial cells (EEs) to acquire a functional and transient ovarian hormone-dependent status, thus allowing blastocyst adhesion. This period, known as the "implantation window", opens 4-5 days after progesterone production or administration and closes 9-10 days after progesterone production or administration. A scientific understanding of the process of endometrial receptivity is the basis for understanding human reproduction and infertility treatment. However, thus far, none of the proposed biochemical indicators of endometrial receptivity have been proven to be clinically useful. The mechanism of regulating endometrial receptivity has been extensively studied in the past two decades. These studies are mainly performed considering morphology and biochemistry; recently, cellular and molecular biological analysis has been applied [1–6]. Most of these studies focus on specific molecules or some members of a specific family, such as integrins, mucins, cytokines, cytoskeleton-related proteins and other molecules [2, 4, 5, 7]. The most widely accepted biochemical marker is the specific pattern of endometrial adhesion molecules, namely, membrane glycoprotein integrin, which mediates adhesion to the extracellular matrix [2, 4]. Additionally, mucin MUC1 is considered a key molecule with important functions during embryo implantation [4]. Furthermore, paracrine and autocrine cytokine systems and factors, such as the interleukin-1 system and leukemia inhibitory factor, control at least part of the adhesion phase of implantation [8, 9]. To date, the regulatory mechanism of endometrial receptivity has not been elucidated, and the key molecules regulating endometrial receptivity have not been found. Therefore, it is necessary to further study the new molecules regulating endometrial receptivity and clarify their regulatory mechanisms.

Small nucleolar RNA (snoRNA) is an important participant in the regulation of gene expression in human cells. The standard functions of box C/D and box H/ACA snoRNAs are posttranscriptional modifications of ribosomal RNA (rRNA)—2-O-methylation and pseudouridine formation, respectively. Defects in ribosome maturation and function can lead to the destruction of life processes, transformation to disease and transformation of normal cells into tumor cells [10–12]. Changes in snoRNA expression may lead to many diseases. The relationship between box C/D RNA and development of neurodegenerative diseases have also been described [13, 14]. Box C/D RNA snord115 may affect the level of serotonin receptor mRNA of 5-HT2CR in the brain [15, 16]. The loss of snord116 snoRNA may contribute substantially to the etiology of PWS [17–19]. The mutation and abnormal expression of snoRNA can lead to various diseases. However, the relationship between snoRNA and endometrial receptivity has not been reported. Additionally, the relationship between snoRNA and endometrial receptivity requires further study, and its role in maintaining endometrial receptivity must be elaborated.

In this study, we used high-throughput sequencing to analyze the expression changes of snoRNA in the endometrium, study the relationship between snoRNA and endometrial receptivity by regulating the expression of snoRNA, and explore the mechanism of snoRNA regulating endometrial receptivity.

## RESULTS

### SNORA75 is upregulated in the mid-secretive phase endometrium

**Figure 1:**
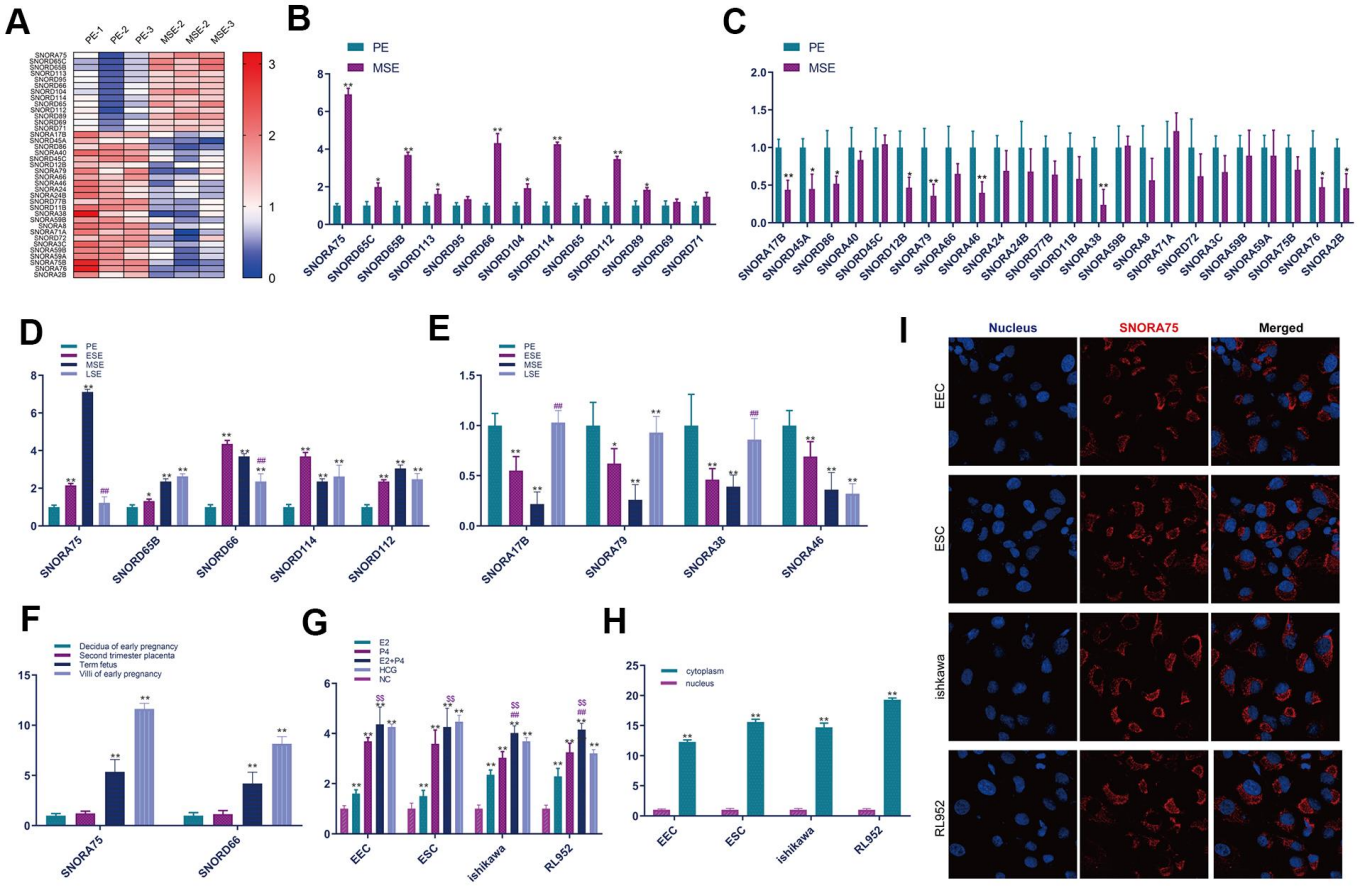
**SNORA75 is upregulated in the mid-secretive phase endometrium.** (**A**) The expression of SNORNA in proliferative (PE) and mid secretory (MSE) endometrium was analyzed by a heatmap. (**B**) The expression of SNORNA in the proliferative (PE) and mid secretory (MSE) endometrium was detected by real-time quantitative fluorescence PCR. (**C**) The expression of SNORNA in proliferative (PE) and mid secretory (MSE) endometrium was detected by real-time quantitative fluorescence PCR. (**D**) Real-time quantitative PCR was used to detect SNORNA expression in different stages of the endometrium. PE: proliferative phase, ESE: early secretory phase, MSE: secretory phase, LSE: late secretory phase. (**E**) Real-time quantitative fluorescence PCR was used to detect snoRNA expression in different stages of the endometrium. (**F**) Real-time quantitative PCR was used to detect the temporal expression of SNORNA in the placenta. (**G**) The expression of SNORA75 in EEC, ESC, Ishikawa and RL952 cells was detected by real-time quantitative fluorescence PCR. (**H**) The expression of SNORA75 in the nucleus and cytoplasm was detected by real-time quantitative fluorescence PCR. (**I**) *In situ* hybridization was used to detect SNORA75 expression in endometrial cells. Blue represents the nucleus, and red represents SNORA75.

To identify snoRNAs associated with endometrial receptivity, we collected the proliferative and secretory endometrium and extracted total RNA for sequencing analysis. Through bioinformatics analysis, based on the criteria of a fold change greater than 2 and a P value less than 0.05, 37 snoRNAs with differential expression were selected. Compared with the proliferation phase, 13 snoRNAs were significantly increased in the secretory metaphase, and 24 were significantly decreased in the secretory metaphase compared with the proliferative phase (Figure 1A). To further verify the sequencing results, we used real-time quantitative fluorescence PCR to verify the sequencing results. The results showed that compared with the proliferative phase (PE) endometrium, the expression of snoRNA in the mid secretory phase (MSE) endometrium was significantly increased as follows: SNORA75, SNORD65C, SNORD 65B, SNORD 113, SNORD 66, SNORD 114, SNORD 112 and SNORD 89 (Figure 1B). The real-time PCR results showed that, compared with the proliferative phase (PE) endometrium, the expression of snoRNA in the mid secretory phase (MSE) endometrium was significantly decreased as follows: SNORA 17B, SNORD 45A, SNORD 86, SNORD 12B, SNORA 79, SNORA 46, SNORA 38, SNORA 76 and SNORA 2B (Figure 1C). Furthermore, we analyzed the expression of snoRNA in different endometrium stages by real-time quantitative PCR. First, we detected significantly increased expression of snoRNA in the MSE group (P < 0.01). The expression of SNORA75 initially increased in the ESE endometrium, with the highest expression in the MSE endometrium, and then decreased in the late stage of endometrial secretion (Figure 1D). The expression of SNORA17B, SNORA79 and SNORA38 initially decreased in the ESE endometrium, with the lowest expression in the MSE endometrium, and increased in late endometrium secretion (Figure 1E). However, the expression of SNORA46 initially decreased from the beginning of the ESE endometrium to the late stage of endometrial secretion (Figure 1E). To detect the temporal expression of snoRNA in the placenta, we collected decidual tissues from early pregnancy, placental tissues from middle pregnancy, and full-term placentas and villi from early pregnancy. The expression of SNORA75 and SNORD66 was significantly increased in the villi from early pregnancy and full-term placenta (Figure 1F). To study the mechanism by which SNORA75 regulates endometrial receptivity, we first detected the effect of reproductive hormones on SNORA75 expression in EEC, ESC, Ishikawa and rl952 cells. EECS, ESC, Ishikawa and RL952 cells were stimulated with estrogen (E2, 10^-8^ M/L), progesterone (P4, 10^-7^ M/L) and hCG (1000 u/L) for 48 hours. E2, P4 and hCG effectively promoted SNORA75 expression in EEC, ESC, Ishikawa and RL952 cells. E2 and P4 showed no superposition effect on SNORA75 expression in EEC and ESC cells, but E2 and P4 had a superposition effect in Ishikawa and RL952 cells (Figure 1G). We detected SNORA75 expression in primary endometrial epithelial cells (EECs), primary endometrial stromal cells (ESCs), Ishikawa cells and RL952 cells by real-time quantitative fluorescence PCR and *in situ* hybridization. The content of SNORA75 in the cytoplasm was significantly higher than that in the nucleus (Figure 1H). *In situ* hybridization was used to detect SNORA75 expression in the nucleus and cytoplasm. SNORA75 was mainly located in the cytoplasm, as its abundance in the nucleus was lower (Figure 1I).

### SNORA75 promotes the proliferation, migration and invasion of endometrial epithelial cells

**Figure 2:**
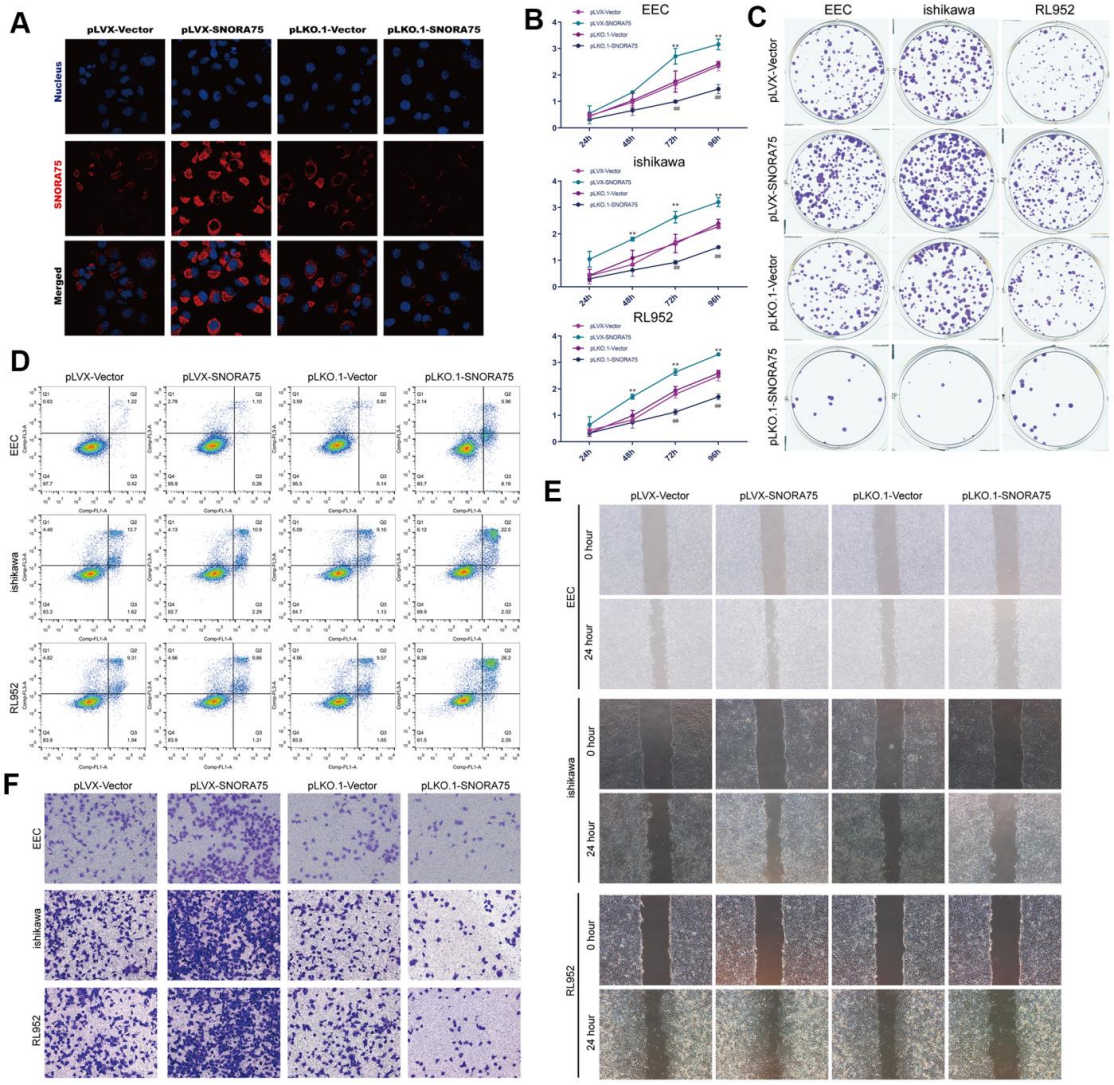
**SNORA75 promotes the proliferation, migration and invasion of endometrial epithelial cells.** (**A**) The expression of SNORA75 in EEC cells was detected by *in situ* hybridization. (**B**) CCK-8 was used to detect the effect of SNORA75 on the proliferation of EEC, Ishikawa and RL952 cells. (**C**) The colony formation assay was used to detect the effect of SNORA75 on the proliferation of EEC, Ishikawa and RL952 cells. (**D**) The apoptosis of EEC, Ishikawa and RL952 cells was detected by flow cytometry. (**E**) The effect of SNORA75 on the migration of EEC, Ishikawa and RL952 cells was detected by the cell scratch assay. (**F**) Transwell assays were used to detect the invasion of EEC, Ishikawa and rl952 cells. "^##^" indicates that, compared with the pLKO.1-vector group, P < 0.01. "^**^" indicates that, compared with the pLVX-vector group, P < 0.01.

To study the effect of SNORA75 on endometrial epithelial cells, we first constructed lentiviruses that overexpressed or knocked down SNORA75 that were transfected into EEC, Ishikawa and RL952 cells. First, we transfected EEC, Ishikawa and RL952 cells with SNORA75 overexpression lentivirus. After 48 hours of transfection, SNORA75 expression in EEC cells was detected by *in situ* hybridization. SNORA75 overexpression increased the content of SNORA75 in the cytoplasm of EECs, while SNORA75 expression in the nucleus was increased. However, the content of SNORA75 in the cytoplasm was not inhibited (Figure 2A). CCK-8 was used to detect the effect of SNORA75 on the proliferation of EEC, Ishikawa and RL952 cells. SNORA75 overexpression in EEC, Ishikawa and RL952 cells effectively promoted the proliferation of EEC, Ishikawa and RL952 cells. Compared with the pLVX-vector group, the cell viability of the pLVX-SNORA75 group was significantly increased at 72 hours and 96 hours, while the knockdown of SNORA75 effectively reduced the activity of EEC, Ishikawa and RL952 cells significantly at 72 hours and 96 hours in the pLKO.1-SNORA75 group compared with that in the pLKO.1-vector group (Figure 2B). Furthermore, we detected the effect of SNORA75 on the proliferation of EEC, Ishikawa and RL952 cells by the clone formation assay. SNORA75 overexpression in EEC, Ishikawa and RL952 cells effectively promoted the proliferation of these cells. Compared with the pLVX-vector group, the number of clones in the pLVX-SNORA75 group was significantly increased, and the knockdown of SNORA75 effectively reduced the proliferation of EEC, Ishikawa and rl952 cells. Compared with the pLKO.1-vector group, the number of cell clones in the pLKO.1-SNORA75 group was significantly reduced (Figure 2C and Supplementary Figure 1A). The effect of SNORA75 on the apoptosis of EEC, Ishikawa and RL952 cells was detected by flow cytometry, revealing that SNORA75 overexpression in EEC, Ishikawa and RL952 cells did not affect the apoptosis of EEC, Ishikawa and rl952 cells. Knockdown of SNORA75 increased the apoptosis rate of EEC, Ishikawa and rl952 cells. Compared with that in the pLKO.1-vector group, the number of apoptotic cells in the pLKO.1-SNORA75 group was significantly increased (Figure 2D and Supplementary Figure 1B). Cell scratch assays showed that SNORA75 overexpression in EEC, Ishikawa and RL952 cells effectively promoted the migration of EEC, Ishikawa and RL952 cells, while the knockdown of SNORA75 in EEC, Ishikawa and RL952 cells effectively inhibited the migration of EEC, Ishikawa and RL952 cells (Figure 2E and Supplementary Figure 1C). Transwell assays showed that compared with the pLVX-vector group, the invasiveness of EEC, Ishikawa and RL952 cells in the plVX-SNORA75 group was significantly increased. Compared with the pLKO.1-vector group, the invasion ability of EEC, Ishikawa and RL952 cells in the pLKO.1-SNORA75 group was significantly decreased (Figure 2F and Supplementary Figure 1D).

### SNORA75 promotes endometrial receptivity

**Figure 3:**
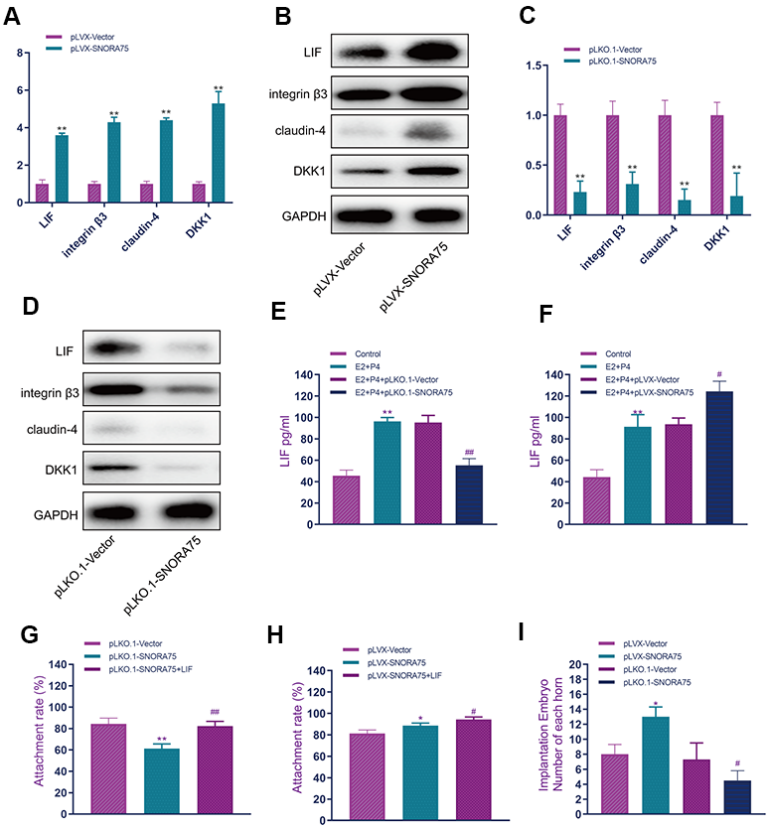
**SNORA75 promotes endometrial receptivity.** (**A**) The effect of SNORA75 overexpression on the expression of receptivity-related factors in EEC cells was detected by real-time quantitative PCR. (**B**) Western blotting was used to detect the effect of SNORA75 overexpression on the expression of receptivity-related factors in EEC cells. (**C**) Real-time quantitative fluorescence PCR was used to detect the effect of SNORA75 knockdown on the expression of receptivity-related factors in EEC cells. (**D**) Western blotting was used to detect the effect of SNORA75 knockdown on the expression of receptivity-related factors in EEC cells. (**E**) The effect of SNORA75 knockdown on LIF expression in EEC cell culture medium was detected by ELISA. (**F**) ELISA was used to detect the effect of SNORA75 overexpression on LIF expression in the culture medium of EEC cells. (**G**) Effect of SNORA75 silencing on the trophoblast adhesion rate. (**H**) Effect of SNORA75 overexpression on the trophoblast adhesion rate. (**I**) Effect of SNORA75 on embryo implantation. "^##^" indicates that, compared with the pLKO.1-vector group, P < 0.01. "^**^" indicates that, compared with the pLVX-vector group, P < 0.01.

To detect the effect of SNORA75 on the expression of receptivity-related factors in EEC cells, we used lentiviral overexpression of SNORA in EEC cells. Real-time quantitative PCR showed that, compared with the control group, SNORA75 overexpression significantly promoted the expression of LIF, integrin3, claudin4 and DKK1 mRNA in EEC cells (Figure 3A). Western blot analysis showed that SNORA75 overexpression significantly promoted the protein expression of LIF, integrin3, claudin4 and DKK1 in EEC cells compared with that in the control group (Figure 3B). Real-time quantitative PCR showed that, compared with the control group, silencing SNORA75 significantly inhibited the mRNA expression of LIF, integrin3, claudin4 and DKK1 in EEC cells (Figure 3C). Western blot analysis showed that, compared with the control group, silencing SNORA75 significantly inhibited the protein expression of LIF, integrin3, claudin4 and DKK1 in EEC cells (Figure 3D). LIF is a secretory protein; thus, we used ELISA to detect the effect of snora75 on LIF expression. Compared with the control group, the expression of LIF in the E2 + P4 + pLKO.1-vector group was significantly increased; compared with the E2 + P4 + pLKO.1-vector group, the expression of LIF in the E2 + P4 + pLKO.1-SNORA75 group was significantly lower than that in the E2 + P4 + pLKO.1-vector group (Figure 3E). Compared with that in the E2 + P4 + pLVX-vector group, the expression of LIF in the E2 + P4 + pLVX-SNORA75 group was significantly higher than that in the E2 + P4 + pLVX-vector group. ELISA showed that SNORA75 overexpression significantly promoted the secretion of LIF in EEC cells (Figure 3F). To evaluate the effect of SNORA75 on the adhesion rate of trophoblasts *in vitro*, we constructed an *in vitro* adhesion model. Compared with the pLKO.1-vector group, the adhesion rate of the pLKO.1-SNORA75 group was significantly decreased. After LIF treatment, the adhesion rate of the pLKO.1-SNORA75 group was reduced (Figure 3G), and SNORA75 overexpression significantly improved the adhesion rate (Figure 3H). To further study the effect of SNORA75 on embryo implantation, we used lentivirus to regulate the expression of SNORA75 in animals, injected lentivirus into the uterine cavity on the third day of pregnancy in mice, and calculated the number of embryos implanted in the uterus on the seventh day of pregnancy. SNORA75 overexpression effectively promoted embryo implantation, while the inhibition of SNORA75 expression significantly inhibited embryo implantation (Figure 3I).

### SNORA75 interacts with miR-146a-3p and regulated the function of miR-146a-3p

**Figure 4:**
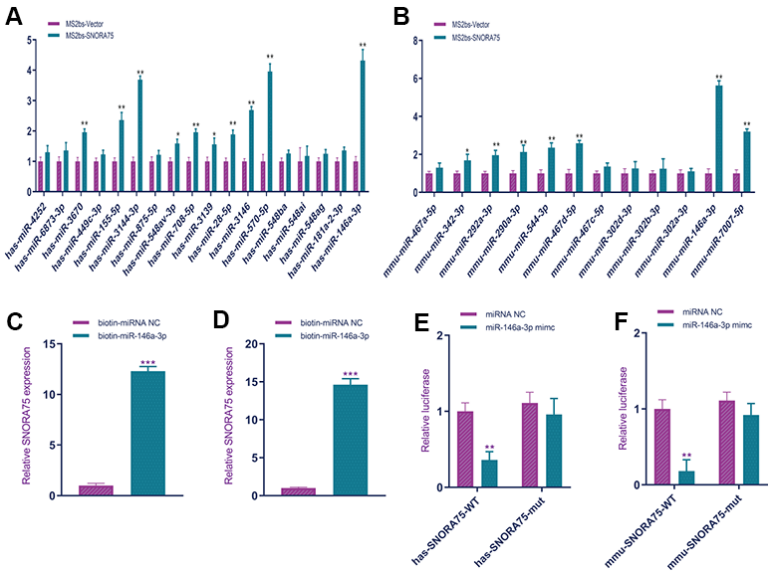
**SNORA75 combined with miR-146a-3p.** (**A**) MS2bs RNA pull-down was used to detect the binding of human SNORA75 and miRNA. (**B**) MS2bs RNA pull-down was used to detect the binding of mouse SNORA75 and miRNA. (**C**) Biotin-mir-146a-3p RNA pull-down was used to detect the binding of hsa-mir-146a-3p to SNORA75. (**D**) Biotin-miR-146a-3p RNA pull-down was used to detect the binding of mmu-mir-146a-3p to SNORA75. (**E**) A luciferase reporter gene was used to detect the binding of miR-146a-3p to hsa-SNORA75.** (F**) A luciferase reporter gene was used to detect the binding of miR-146a-3p to hsa-SNORA75. "^##^" indicates that, compared with the pLKO.1-vector group, P < 0.01. "^**^" indicates that, compared with the pLVX-vector group, P < 0.01.

Non-coding RNAs can regulate cell function in various ways, and the ceRNA mechanism is a common mechanism. First, we used bioinformatics software to analyze the miRNAs that can bind to SNORA75. The software predicted that human-derived SNORA75 could bind to 18 miRNAs, while software predicted that mouse-derived SNORA75 could bind to 13 miRNAs. According to the prediction of the software, we used MS2b RNA pulldown to verify the binding of SNORA75 to miRNA. Using the MS2bs RNA pulldown assay, we found that human SNORA75 bound to hsa-miR-3670, hsa-miR-155-5p, hsa-miR-3144-3p, hsa-miR-548av-3p, hsa-miR-708-5p, hsa-miR-3139, hsa-miR-28-5p, hsa-miR-3146, hsa-miR-570-5p, and hsa-miR-146a-3p; the binding amount of hsa-miR-146a-3p was higher (Figure 4A). Additionally, we analyzed the binding of mouse SNORA75 to miRNA. Using MS2bs RNA pulldown detection, we found that human SNORA75 bound to mmu-miR-342-3p, mmu-miR-292a-3p, mmu-miR-544-3p, mmu-miR-467d-5p, mmu-miR-146a-3p, and mmu-miR-7007-5p, among which hsa-miR-146a-3p was more abundant (Figure 4B). To further verify the binding of SNORA75 and miR-146a-3p, we constructed biotin-labeled miR-146a-3p (biotin-miR-146a-3p). Using biotin-miR-146a-3p RNA pulldown, we detected the binding of miR-146a-3p and SNORA75 in human and mouse EEC cells, respectively, and human SNORA75 binding to hsa-miR-146a-3p (Figure 4C). Similarly, mouse-derived SNORA75 also bound to mmu-miR-146a-3p (Figure 4D). The wild-type and mutant sequences of miR-146a-3p binding to SNORA75 were cloned into the psicheck2 luciferase reporter gene vector. miR-146a-3p effectively inhibited the luciferase reporter gene activity of wild-type hsa-SNORA75-psicheck2 but did not affect the activity of the mutant hsa-SNORA75-psicheck2 luciferase reporter gene (Figure 4E). Additionally, miR-146a-3p effectively inhibited the luciferase reporter gene activity of wild-type hsa-SNORA75-psicheck2 but did not affect the activity of the mutant hsa-SNORA75-psicheck2 luciferase reporter gene (Figure 4F).

### MiR-146a-3p suppresses endometrial receptivity

**Figure 5:**
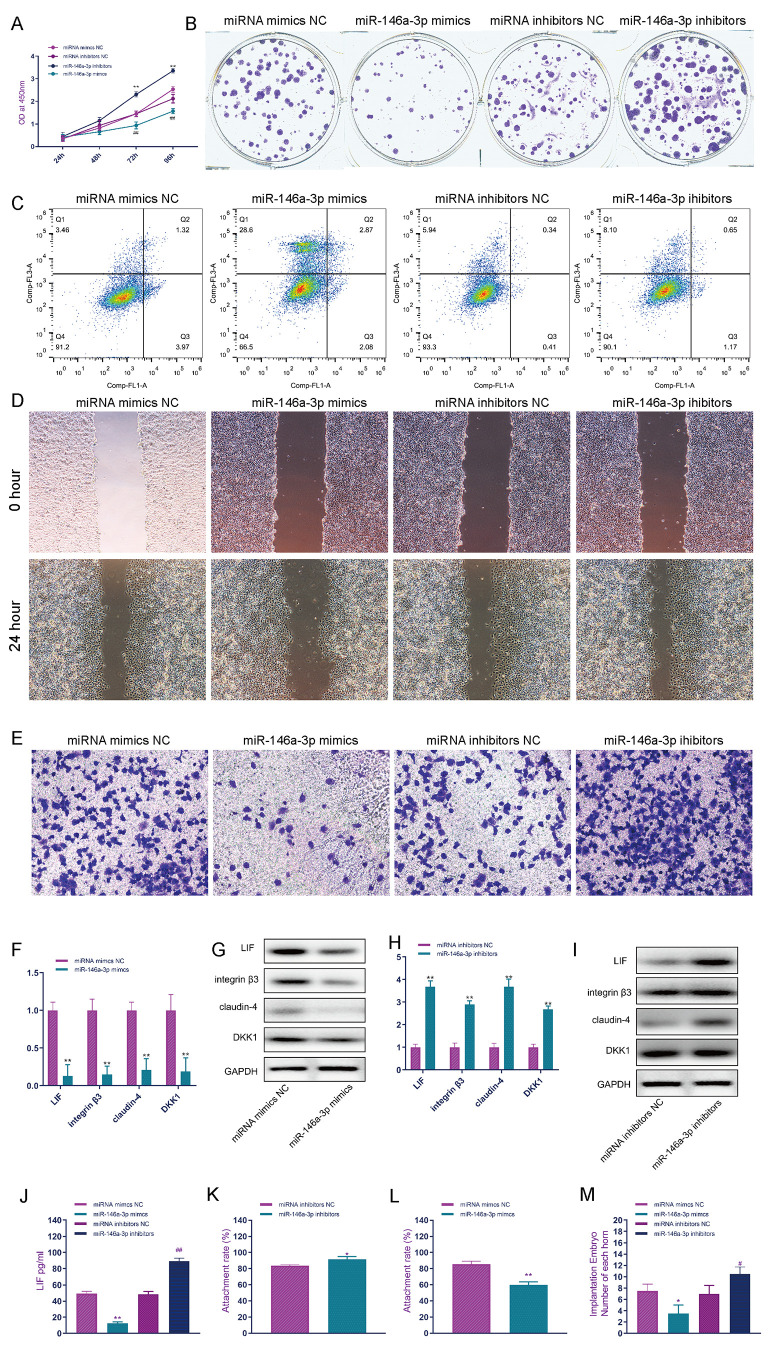
**MiR-146a-3p suppresses endometrial receptivity.** (**A**) CCK-8 was used to detect the effect of miR-146a-3p on the proliferation of EEC cells. (**B**) The effect of miR-146a-3p on the proliferation of EEC cells was detected by the clone formation assay. (**C**) The effect of miR-146a-3p on the apoptosis of EEC cells was detected by flow cytometry. (**D**) The scratch assay was used to detect the effect of miR-146a-3p on the invasion of EEC cells. (**E**) Transwell assays were used to detect the effect of miR-146a-3p on the invasion of EEC cells. (**F**) The effect of miR-146a-3p overexpression on the expression of receptivity-related factors in EEC cells was detected by real-time quantitative PCR. (**G**) Western blotting was used to detect the effect of miR-146a-3p on the expression of receptivity-related factors in EEC cells. (**H**) Real-time quantitative PCR was used to detect the effect of miR-146a-3p knockdown on the expression of receptivity-related factors in EEC cells. (**I**) Western blotting was used to detect the effect of miR-146a-3p knockdown on the expression of receptivity-related factors in EEC cells. (**J**) The effect of knockdown of miR-146a-3p on LIF expression in EEC cell culture medium was detected by ELISA. (**K**) Effect of miR-146a-3p overexpression on the trophoblast adhesion rate. (**L**) Effect of miR-146a-3p inhibition on the trophoblast adhesion rate. (**M**) Effect of miR-146a-3p on embryo implantation. "^*^" indicates P < 0.05, "^**^" indicates P < 0.01, and "^***^" indicates P < 0.001.

CCK-8 detection results showed that miR-146a-3p overexpression in EEC cells effectively inhibited the proliferation of EECs, and inhibiting the function of miR-146a-3p effectively improved the activity of EEC cells (Figure 5A). The effect of miR-146a-3p on the proliferation of EEC cells was detected by the clone formation assay. miR-146a-3p overexpression in EEC cells effectively inhibited the proliferation of EEC cells, and the knockdown of miR-146a-3p effectively promoted the proliferation of EEC cells (Figure 5B and Supplementary Figure 2A). Flow cytometry showed that miR-146a-3p overexpression in EEC cells significantly promoted the apoptosis of EEC cells. Compared with the number of apoptotic cells in the miRNA mimic NC group, that in apoptotic cells in the miR-146a-3p mimic group was significantly increased, while the apoptosis rate of EEC cells was not affected by miR-146a-3p knockdown (Figure 5C and Supplementary Figure 2B). The cell scratch assay showed that miR-146a-3p overexpression in EEC cells effectively inhibited the migration of EEC cells, while the knockdown of miR-146a-3p in EEC cells effectively promoted the migration of EEC cells (Figure 5D and Supplementary Figure 2C). Transwell assays showed that, in the miR-146a-3p mimic group, the invasion ability of EEC cells was significantly decreased and that of the miR-146a-3p inhibitor group was significantly increased (Figure 5E and Supplementary Figure 2D). MiR-146a-3p overexpression significantly inhibited the mRNA expression of LIF, integrin3, claudin4 and DKK1 in EEC cells (Figure 5F). Western blot analysis showed that miR-146a-3p overexpression significantly inhibited the expression of LIF, integrin3, claudin4 and DKK1 in EEC cells (Figure 5G). Silencing miR-146a-3p significantly promoted the expression of LIF, integrin3, claudin4 and DKK1 mRNA in EEC cells (Figure 5H). Western blot analysis showed that miR-146a-3p silencing significantly promoted the protein expression of LIF, integrin3, claudin4 and DKK1 in EEC cells (Figure 5I). ELISA showed that knockdown of miR-146a-3p significantly promoted the secretion of LIF in EEC cells (Figure 5J), and snora75 overexpression significantly promoted the secretion of LIF in EEC cells (Figure 5K). To investigate the effect of miR-146a-3p on the adhesion rate of trophoblasts *in vitro*, we constructed an *in vitro* adhesion model. The adhesion rate of the miR-146a-3p mimic group was significantly lower than that of the miRNA mimic NC group (Figure 5L), and inhibition of miR-146a-3p significantly improved the adhesion rate (Figure 5K). To further study the effect of miR-146a-3p on embryo implantation, we used miR-146a-3p miRNA mimics and inhibitors to regulate the expression of miR-146a-3p in animals. MiR-146a-3p overexpression inhibited the implantation of embryos, and inhibition of miR-146a-3p expression significantly promoted embryo implantation (Figure 5M).

### SNORA75 promotes ZNF23 expression by modulating miR-146a-3p

**Figure 6:**
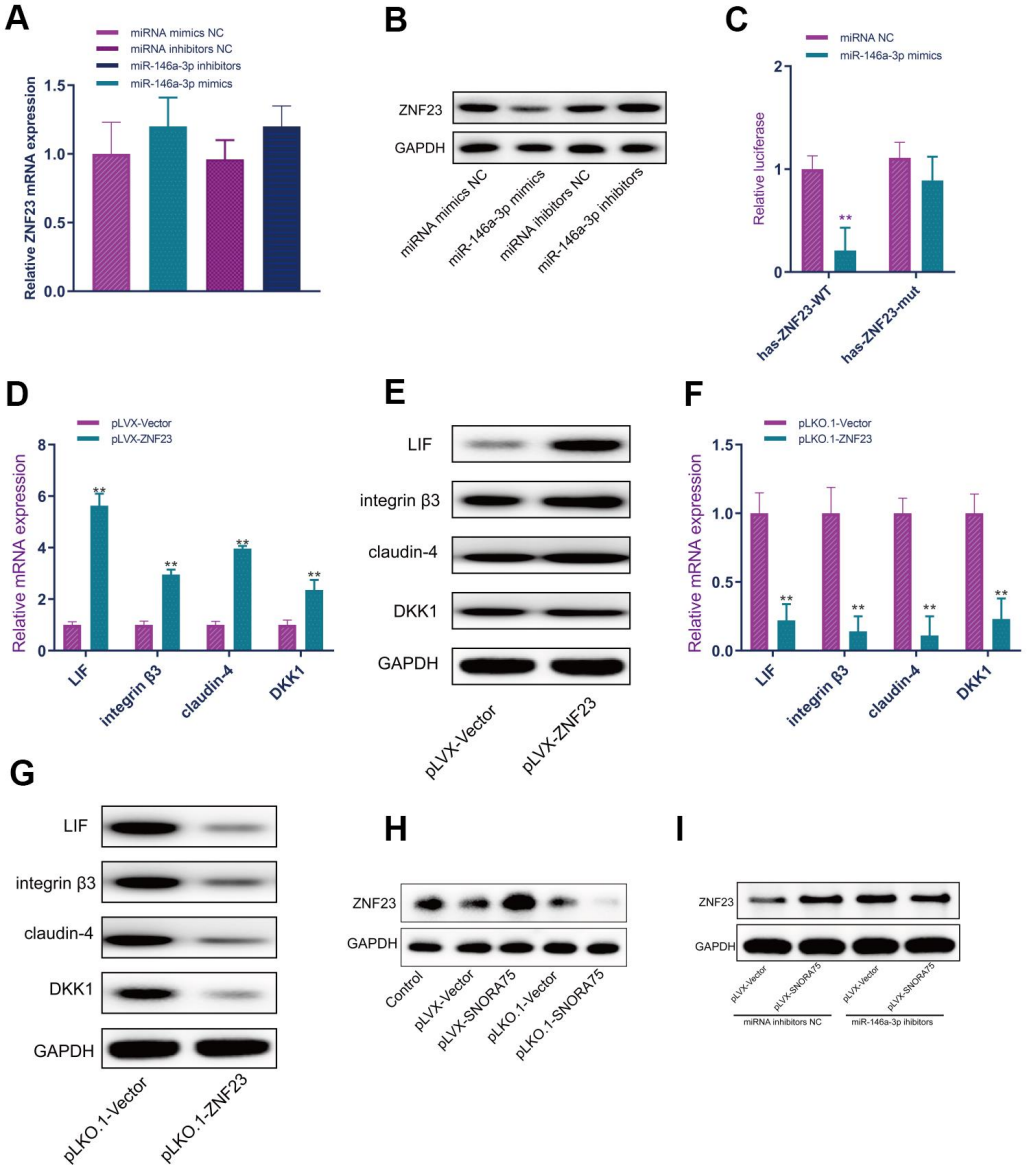
**SNORA75 promotes ZNF23 expression by modulating miR-146a-3p.** (**A**) The effect of miR-146a-3p on ZNF23 mRNA expression was detected by real-time quantitative fluorescence PCR. (**B**) The effect of miR-146a-3p on ZNF23 protein expression was detected by Western blotting. (**C**) The binding of miR-146a-3p to hsa-ZNF23 was detected by the luciferase reporter assay. (**D**) The effect of ZNF23 overexpression on the expression of receptivity-related factors in EEC cells was detected by real-time quantitative PCR. (**E**) Western blotting was used to detect the effect of ZNF23 overexpression on the expression of receptivity-related factors in EEC cells. (**F**) The effect of ZNF23 knockdown on the expression of receptivity-related factors in EEC cells was detected by real-time quantitative fluorescence PCR. (**G**) Western blotting was used to detect the effect of ZNF23 knockdown on the expression of receptivity-related factors in EEC cells. (**H**) ZNF23 expression was detected by Western blotting. (**I**) ZNF23 expression was detected by Western blotting. "^**^" indicates P < 0.01.

MiR-146a-3p mimics and inhibitors were used to overexpress and inhibit miR-146a-3p expression in EEC cells, and the results of real-time PCR showed that miR-146a-3p overexpression and inhibition did not affect ZNF23 mRNA expression in EEC cells (Figure 6A). Western blot analysis showed that miR-146a-3p overexpression significantly inhibited the expression of ZNF23 protein, and miR-146a-3p inhibition significantly promoted ZNF23 expression (Figure 6B). To further verify the accuracy of the binding site, we cloned the wild-type and mutant sequences of the binding site of miR-146a-3p with ZNF23 into the psicheck2 luciferase reporter gene vector. MiR-146a-3p effectively inhibited the luciferase reporter gene activity of wild-type hsa-ZNF23-psicheck2 in human EEC cells but did not affect the activity of the mutant hsa-znf23-psicheck2 luciferase reporter gene (Figure 6C). To detect the effect of ZNF23 on the expression of receptivity-related factors in EEC cells, a ZNF23 overexpression lentivirus was used to overexpress ZNF23 in EEC cells. Real-time quantitative PCR showed that ZNF23 overexpression significantly promoted the mRNA expression of LIF, integrin3, claudin4 and DKK1 in EEC cells (Figure 6D). Compared with the control group, ZNF23 overexpression significantly promoted the expression of LIF, integrin3, claudin4 and DKK1 in EEC cells, as detected by Western blotting (Figure 6E). ZNF23 was silenced in EEC cells by ZNF23 knockdown lentivirus. Silencing ZNF23 significantly inhibited the mRNA expression of LIF, integrin3, claudin4 and DKK1 in EEC cells (Figure 6F). Western blot analysis showed that ZNF23 silencing significantly inhibited the protein expression of LIF, integrin3, claudin4 and DKK1 in EEC cells (Figure 6G). Western blot analysis showed that SNORA75 overexpression significantly promoted ZNF23 expression, and SNORA75 inhibition significantly inhibited ZNF23 expression (Figure 6H). Western blot analysis showed that when the function of miR-146a-3p was inhibited, the SNORA75-mediated promotion of ZNF23 expression was lost, indicating that this effect was miR-146a-3p dependent (Figure 6I).

## DISCUSSION

Endometrial receptivity is a complex phenomenon that plays an important role in infertility. Although embryo quality can be evaluated for successful implantation, endometrial receptivity remains an unknown factor. With the latest development of sequencing technology, genomics, lipomics and proteomics analysis of the endometrium may provide the "best" evaluation tools and standards for evaluating receptivity in the near future.

Post-transcriptional modification of RNA and the control of mRNA stability and translation are important components of gene expression regulation in human cells. Small nucleolar RNAs and their functional fragments play important roles in these processes: they direct the nucleotide modification of rRNA and snRNA, influence the alternative splicing of complementary pre mRNA, and control the translation and stability of mRNA through a RISC-dependent pathway. The destruction of snoRNA expression may be caused by external factors and intracellular signaling cascades, leading to physiological changes at the cellular level, organ dysfunction and various diseases. The structure, expression pattern and intracellular localization of snoRNAs have regulatory significance and are considered diagnostic markers of pathology. Further understanding of snoRNA expression and its functional mechanism will provide new possibilities for developing human disease diagnosis systems and new treatment methods. We found that SNORA75 has a strong correlation with endometrial receptivity and can be used as an evaluation index of endometrial receptivity.

MiRNAs are small non-encoding RNAs that play a role in RNA silencing and post-transcriptional regulation of gene expression. [20, 21]. Recent studies have shown that miRNAs are expressed in plasma, serum and other body fluids [22, 23]. Almost all types of cells can secrete miRNA, and the concentration of extracellular miRNA is related to the physiological and pathological conditions of the human body [24]. Some extracellular miRNAs may also be involved in intercellular communication, and the implantation process involves the coordination of complex regulatory systems between the embryo and maternal uterus [25, 26]. Evidence from peri-implantation miRNA regulation of embryonic development and uterine function suggests that miRNA plays an important role in this process. Additionally, the discovery of extracellular miRNAs in the uterine cavity fluid (ULF) and embryo culture medium suggests the need to explore new potential miRNAs, particularly in assisted reproduction. Based on their conservation, stability, sensitivity and accessibility, extracellular miRNAs are considered valuable non-invasive biomarkers for evaluating embryo viability and endometrial receptivity. Implantation is a complex process that requires the simultaneous development of a viable embryo and a pregnant endometrium. MiRNAs act as regulators of gene expression and are actively involved in regulating embryonic development, endometrial function and embryo mother communication. We found that miR-146a-5p strongly correlates with endometrial receptivity, which can be used as an evaluation index of endometrial receptivity.

## CONCLUSIONS

Compared with snoRNA expression in the proliferative phase, that in the secretory phase changed significantly. The highest SNORA75 expression was found in the middle secretory phase of the endometrium. E2, P4 and hCG can induce SNORA75 expression in endometrial cells. SNORA75 promotes the proliferation, migration and invasion of endometrial cells. SNORA75 was targeted to regulate the function of miR-146a-3p, and miR-146a-3p regulated the function of endometrial cells by regulating ZNF23 expression. LIF stimulates the endometrium to release exosomes rich in SNORA75. Through the SNORA75/miR-146a-3p/ZNF23/LIF signaling pathway, SNORA75 regulates the function of endometrial cells and promotes endometrial receptivity.

## MATERIALS AND METHODS

### Collection of endometrial epithelial tissue specimens

The specimens were collected from the First Affiliated Hospital of Zhengzhou University, with approval from the hospital’s ethics committee, and signed informed consent was obtained. The sample collection criteria were as follows: 1) an age range of 25-35 years a menstrual cycle of 25-35 days; 2) reproductive system diseases such as ovarian cysts, uterine fibroids, infertility due to fallopian tube factors, and benign cervical intraepithelial lesions; 3) no laparotomy, hysteroscopy or colposcopy surgery; 4) no acute and chronic inflammation of the reproductive system; 5) no reproductive system tumors; 6) no chronic diseases such as diabetes, hepatitis and thyroid diseases; and 7) no hormone drugs taken 3 months before sampling. The endometrium was aspirated using an endometrial straw.

### Isolation and culture of endometrial epithelial cells

Fresh endometrial tissues were collected and washed with PBS containing antibiotics to remove blood stains and residual tissues. Fresh endometrial tissue was transferred into DMEM containing antibiotics and no serum, and the tissue was cut into 1-mm^3^ tissue blocks. The final concentration of collagenase type II was 0.2% (w/V), and the contents were mixed well, placed in a 37 ° C incubator, shaken at 250 rpm for 1 hour, and then removed. The digestive juice was filtered through a 100-mesh screen and then filtered with a 400-mesh screen. Cells that did not pass the 400-mesh screen were cultured in DMEM containing 10% fetal bovine serum. The cells were inoculated in a 10-cm culture dish, which was incubated overnight at 37° C in 5% CO_2_ and saturated humidity. When the cells reached 70% healing, they were subcultured.

### Real-time quantitative fluorescence PCR detection

Two hundred milligrams of fresh endometrial tissue was weighed. Next, 1 ml of TRIzol solution was added, followed by homogenization of the tissue with a homogenizer, the addition of 200 μl of chloroform, and rigorous shaking for 30 seconds. The samples were allowed to stand at room temperature for 15 minutes and then were subjected to centrifugation at 12000 rpm for 15 minutes at 4° C. The supernatants were collected, and then an equal volume of cold isopropyl alcohol solution was added. The samples were allowed to stand for 15 minutes at 4° C and then were subjected to centrifugation at 12000 rpm for 10 minutes. The supernatants were discarded, and 1 ml of cold 75% ethanol solution was added to the precipitates. The mixture was then centrifuged at 12000 rpm for 10 minutes at 4° C. The supernatants were discarded, and then 1 ml of cold 75% ethanol solution was added to the precipitates, followed by centrifugation at 12000 rpm at 4° C for 10 minutes, air drying in a ventilated place, and the addition of 20 μl of DEPC-treated water. According to the instructions of the RT kit, RNA was reverse transcribed into cDNA. Quantitative PCR was performed according to the instructions of the real-time quantitative fluorescence PCR kit. The relative expression value was calculated using the 2 ^-ΔΔ CT^ value.

### Cell viability detection

Cells were seeded into each well of a 96-well plate at a density of 1000 cells per well. After cell inoculation, 10 μl of the CCK-8 reagent was added at different time points. The cells were incubated at 37° C for 2 hours. The absorbances at 450 nm and 650 nm were detected.

### Detection of apoptosis by flow cytometry

The cells were digested with trypsin and centrifuged at 1000 rpm and 4° C for 5 minutes. The supernatant was discarded, and then the cells were resuspended in 100 μl of binding buffer. Next, 5 μl of FITC-annexin V solution was added, and then the contents were thoroughly mixed and incubated at 4° C for 15 minutes. One microliter of PI was added, and then the contents were mixed well and incubated at 4° C for 5 minutes. Next, 400 μl of binding buffer was added, and the contents were mixed well. Flow cytometry was used to detect apoptosis.

### Cell cycle detection

The cells were digested with trypsin and centrifuged at 1000 rpm and 4° C for 5 minutes. The supernatant was discarded, 70% ethanol was added to the suspension, and the contents were fixed at 4° C overnight. The samples were then centrifuged at 1000 rpm at 4° C for 5 minutes. The supernatant was discarded, and then the pellet was resuspended in PBS ethanol, followed by centrifugation at 1000 rpm at 4° C for 5 minutes. The supernatants were discarded and then were incubated with cell cycle staining buffer for 15 minutes. Flow cytometry was used to detect apoptosis.

### Clone formation detection

The cells were seeded into a 6-well plate at a density of 250 cells per well, and the medium was changed every 3 days. After two weeks of culture, 4% PFA was used for fixation for 15 minutes and then the cells were stained with crystal violet for 30 minutes. The samples were then washed twice with PBS and photographed, and the number of clones was recorded.

### Western blot analysis

Two volumes of autoclaved water and 1 mm PMSF were added to each volume of cell precipitation. Next, ultrasonic treatment was performed on ice, waiting 30 seconds between each ultrasound. The samples were then centrifuged in a microcentrifuge at 13000 rpm for 5 minutes and subjected to 10% SDS gel electrophoresis for protein separation, nitrocellulose membrane immersed in wet printing transfer buffer for at least 15 minutes, to remove electrophoretic salts and detergent. The film was transferred at 100 V for 1 hour. The membrane was sealed in 5% skim milk TBST solution for at least 30 minutes. The first antibody was incubated at 4° C overnight, the second antibody was incubated at room temperature for 1 hour, washed with TBST at room temperature 3 times, and then exposed.

### Statistical analysis

Each experiment was verified three times, and all the data were expressed as means ± standard deviation. The difference between the two groups was analyzed by two-tailed Student's t-test. One-way ANOVA was used for statistical analysis of multiple groups of data. A p value < 0.05 indicated a significant difference. “ *” indicates P < 0.05, “* *” indicates P < 0.01, and “***” indicates P < 0.001.

## Supplementary Material

Supplementary Figures
