# Supplementary material for: Nucleolar small molecule RNA SNORA75 promotes endometrial receptivity by regulating the function of miR-146a-3p and ZNF23
Source: Aging (Albany NY). 2021 May 24;13(11):14924–39. doi: 10.18632/aging.203007 (PMC8221328; doi:10.18632/aging.203007)
Supplement: Supplementary Figures [file aging-13-203007-s001.pdf]

SUPPLEMENTARY FIGURES

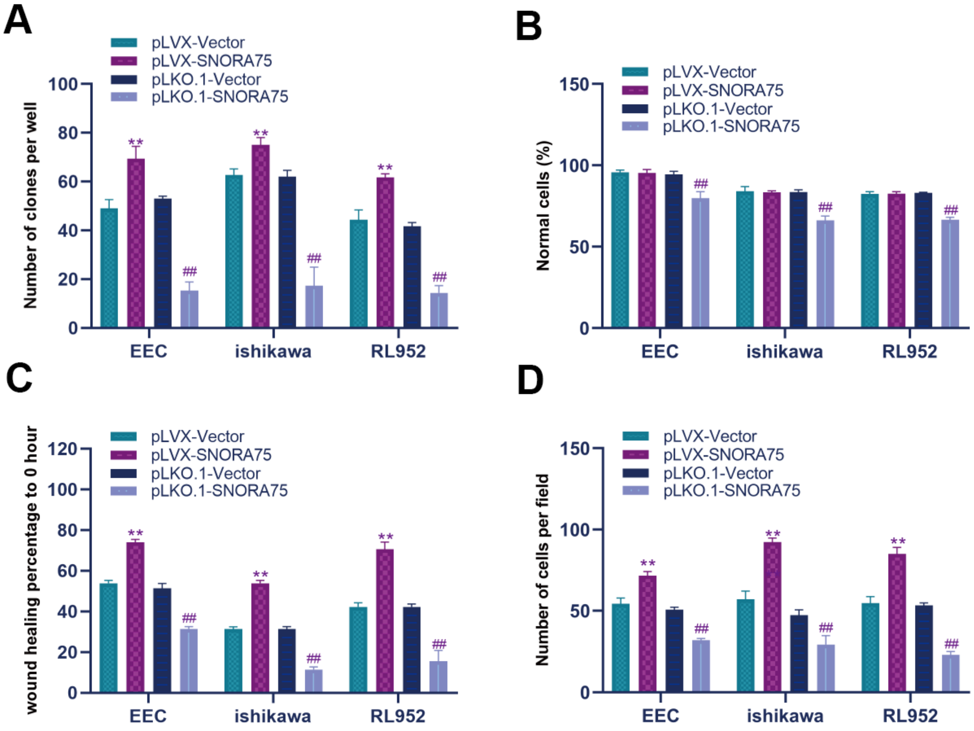

Supplementary Figure 1. Statistical analyses the effect of SNORA75 on the proliferation, migration and invasion of endometrial epithelial cells. (**A**) Statistical analyses the colony formation of EEC, Ishikawa and RL952 cells. (**B**) Statistical analyses the apoptosis of EEC, Ishikawa and RL952 cells. (**C**) Statistical analyses the migration of EEC, Ishikawa and RL952 cells. (**D**) Statistical analyses the invasion of EEC, Ishikawa and RL952 cells. "##" indicates that, compared with the pLKO.1-vector group, $P < 0.01$. "**" indicates that, compared with the pLVX-vector group, $P < 0.01$.

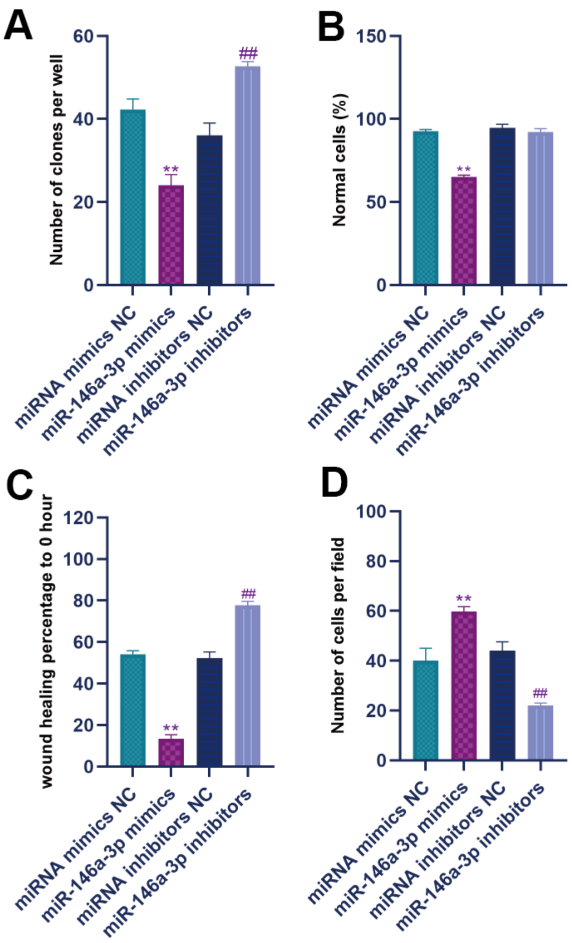

Supplementary Figure 2. Statistical analyses the effect of miR-146a-3p on the proliferation, migration and invasion of endometrial epithelial cells. (**A**) Statistical analyses the colony formation of EEC cells. (**B**) Statistical analyses the apoptosis of EEC cells. (**C**) Statistical analyses the migration of EEC cells. (**D**) Statistical analyses the invasion of EEC cells. "##" indicates that, compared with the pLKO.1-vector group, P < 0.01. "**" indicates that, compared with the pLVX-vector group, P < 0.01.
